# 7,8-Dihydroxyflavone Attenuates Alcohol-Related Behavior in Rat Models of Alcohol Consumption via TrkB in the Ventral Tegmental Area

**DOI:** 10.3389/fnins.2020.00467

**Published:** 2020-05-19

**Authors:** Xin-Xin Li, Tao Yang, Na Wang, Li-Li Zhang, Xing Liu, Yan-Min Xu, Qing Gao, Xiao-Feng Zhu, Yan-Zhong Guan

**Affiliations:** Department of Physiology and Neurobiology, Mudanjiang Medical University, Mudanjiang, China

**Keywords:** 7, 8-Dihydroxyflavone, TrkB, BDNF, ventral tegmental area, alcohol-related behavior

## Abstract

Alcohol use disorder (AUD) is a ubiquitous substance use disorder in the world, of which neural mechanisms remain unclear. Alcohol consumption induces neuro-adaptations in the dopaminergic system originating from the ventral tegmental area (VTA), an important brain region for the reward function in AUD. Endogenous brain-derived neurotrophic factor (BDNF)-TrkB implicated in the development of neuroplasticity, including long-term potentiation of GABAergic synapses (LTP_*GABA*_). We previously found that ethanol blocks LTP_*GABA*_ in the VTA, either *in vivo* or *in vitro*. 7,8-dihydroflavone (7,8-DHF), a BDNF-mimicking small compound, was recently found to penetrate the blood–brain barrier to mimic the biological role of BDNF-TrkB. In this study, we demonstrate that repeated ethanol consumption (including intermittent and continuous ethanol exposure) results in low expression of BDNF in rat VTA. The amount of ethanol intake enhances significantly in rats with intermittent ethanol exposure after 72 h abstinence. Withdrawal signs emerge in rats with continuous ethanol exposure within 3 days after abstinence. Using behavioral tests, intraperitoneal injection of 7,8-DHF can reduce excessive ethanol consumption and preference as well as withdrawal signs in rats with repeated ethanol exposure. Interestingly, microinjection of K252a, an antagonist of TrkB, into the VTA blocks the effects of 7,8-DHF on ethanol-related behaviors. Furthermore, direct microinjection of BDNF into the VTA mimics the effect of 7,8-DHF on ethanol related behaviors. Taken together, 7,8-DHF attenuates alcohol-related behaviors in rats undergoing alcohol consumption via TrkB in the VTA. Our findings suggest BDNF-TrkB in VTA is a part of regulating signals for opposing neural adaptations in AUD, and 7,8-DHF may serve as a potential candidate for treating alcoholism.

## Introduction

The main characteristic of alcohol use disorder (AUD) is the consumption of large quantities of alcohol despite the negative consequences (World Health Organization [WHO], 2014; Ron and Barak, 2016). In 2015, the estimated ratio of the number of occurrences of AUD among the adult population was 18.3% for heavy episodic ethanol use (Peacock and Leung, 2018), and AUD causes tremendous personal and socioeconomic burdens (Volkow et al., 2016). AUD is characterized by a strong urge to consume alcohol, unsuccessful attempts to limit alcohol intake, and emergence of withdrawal signs during no ethanol consumption (Logrip et al., 2015). Consumption of ethanol is regulated by neurochemical systems within specific neural circuits (McGough et al., 2004), but accurate endogenous systems that may counteract and therefore relieve alcohol consumption are unknown (McGough et al., 2004).

Alcohol consumption induces neuroadaptations in the brain reward system (i.e., the mesolimbic dopaminergic system; Kauer and Malenka, 2007), which consists of dopamine neurons in the ventral tegmental area (VTA) projecting to neural substrates involved in reward processing, such as the nucleus accumbens and the medial prefrontal cortex (mPFC) (Kauer and Malenka, 2007; Russo and Nestler, 2013). It is accepted that the VTA is an important brain region for the reward function in AUD (Gonzales et al., 2004; Deehan et al., 2013), although it should not to be ignored that ventral pallidum projections to hypothalamus and/or subthalamic nucleus are strongly implicated in reinstatement and reacquisition of alcohol seeking (Prasad and McNally, 2016). Ethanol action in the VTA may induce neuroadaptation, leading to a relative increase in the rewarding effects of ethanol (You et al., 2018). Previous evidence indicates that long-term potentiation of GABAergic synapses (LTP_*GABA*_) (Nugent et al., 2007) could be induced in VTA, and we found that ethanol impairs LTP_*GABA*_ in the VTA either *in vivo* or *in vitro* (Guan and Ye, 2010). Nonetheless, the function of LTP_*GABA*_ in VTA is still unknown. Brain-derived neurotrophic factor (BDNF), a member of the neurotrophic protein family, and its receptor, tropomyosin receptor kinase B (TrkB), are extensively expressed in the central nervous system (Chao, 2003), including the VTA, and are thought to be involved in neurotransmitter release (Chao, 2003) as well as development of neuroplasticity, including LTP_*GABA*_ (Kuczewski et al., 2008; Panja and Bramham, 2014). BDNF-TrkB signaling not only regulates GABAergic transmission in the rat supraoptic nucleus (Ohbuchi et al., 2009), but also is required for the induction of LTP_*GABA*_ in visual cortical pyramidal neurons and hippocampus (Inagaki et al., 2008; Lu et al., 2010). The signaling pathways triggered by BDNF-TrkB in VTA may underlie some physiological functions including behavior effects of ethanol and natural reward (Nikulina et al., 2014).

An increasing number of literatures suggest a role of BDNF in alcohol addiction (Davis, 2008). A prolonged voluntary ethanol consumption produces a significant decrease in BDNF expression in the mPFC (Darcq et al., 2015), which was directly related to the amount of ethanol consumption in mice (Darcq et al., 2015). Increasing BDNF level in the mPFC can selectively reduce excessive ethanol intake in ethanol depending mice (Haun et al., 2018). Inhibition of TrkB in the dorsolateral striatum increases ethanol consumption and preference (Jeanblanc et al., 2009). However, a correlation between BDNF-TrkB in VTA and alcohol-consuming behavior remains elusive. BDNF is part of a macromolecular substance, which is difficult to cross the blood–brain barrier (BBB) (Zuccato and Cattaneo, 2009). 7,8-dihydroxyflavone (7,8-DHF), a small potent agonist of TrkB, can go across the BBB after Systemic administration to mimic the biological role of BDNF-TrkB (Jang et al., 2010). The discovery of 7,8-DHF solves this problem, and this molecule has been recently validated in diverse biochemical and cellular systems (Liu et al., 2016). It was found that 7,8-DHF could interfere with depression (Zhang et al., 2014) and cognitive function deficits (Tan et al., 2016) effectively in animal models. AUDs are usually combined with psychiatric disorders, which may be the risk factors of AUDs and brings about more serious outcomes for alcoholics (Yang et al., 2018). To this day, a report on the effects of 7,8-DHF in AUD has not been found.

In this study, we report that repeated alcohol exposure leads to a low expression of BDNF in the VTA. In addition, 7,8-DHF attenuates alcohol-related behavior in alcohol-consuming rats via the TrkB in the VTA, and those effects of 7,8-DHF could be mimicked by direct infusion of BDNF into the VTA.

## Materials and Methods

### Reagents and Drug Preparation

All drugs, such as BDNF, 7,8-DHF, K252a, and dimethyl sulfoxide (DMSO), were purchased from Sigma-Aldrich (Harbin, China). Ethanol solutions were prepared from 99% (v/v) ethyl alcohol (Oceanpak, Shenzhen, China) using tap water. 7,8-DHF was diluted with DMSO, and administered through intraperitoneal injection. BDNF or K252a dissolved in artificial cerebrospinal fluid were microinjected into VTA.

### Animals

Adult, male Sprague-Dawley (SD) rats (Harbin, China), weighing 180–200 g at the beginning of experiment were separately housed in ventilated Plexiglas cages to maintain a stable temperature (22 ± 2°C). The rats were allowed to acclimatize to the separate housing conditions in advance, and they had free access to food and water. All rats in this study were housed under a 12 h light/dark cycle with lights off at 20:00 (8 p.m.). Operations were implemented between 9:00 and 16:00 h. All procedures were approved by the guidelines in the National Institutes of Health Guide for Care and Use of Laboratory Animals and the Institutional Animal Care and Use Committee of the Mudanjiang Medical University.

### Intermittent Access to Ethanol

Intermittent access to ethanol using a two-bottle choice ethanol consumption procedure (IA2BC) was performed as previous description (Carnicella et al., 2014). Briefly, each rat was given access to one bottle of 20% (v/v) ethanol without sweeteners and one bottle of water. 24 h later, the alcohol bottle was replaced with another water bottle available for the next 24 h (Flores-Bastias et al., 2019). The placement of the alcohol bottle was alternated between alcohol consuming sessions in order to control for side preference. Ethanol consumption was determined by calculating grams of alcohol consumed per kilogram of body weight before and after 24 h of access, and preference for ethanol was calculated as the amount of ethanol consumed as a percentage of the total fluid consumption: % preference = mL of ethanol/(mL of ethanol + mL of water)^∗^100% (Li et al., 2012). Rats in control group had free access to water until the end of the experiment.

### Chronic Alcohol Exposure

This procedure of chronic alcohol exposure was carried out like description by Turchan et al. (1999), with some modifications. The experimental rats were given a 1% (v/v) alcohol as the only liquid source on the first day. After 2 days of exposure, the 1% (v/v) ethanol changed to a 3% (v/v) ethanol solution. The 3% (v/v) ethanol solution changed to 6% (v/v) ethanol solution on sixth day, a choice that lasted for 22 days. The ethanol solution was refreshed at 9:00 h each day. The rat weights were recorded daily, and the alcohol consumption was calculated and described as grams per kilogram body weight per 24 h. Rats in the control group were provided with water for 28 days.

### Ethanol Withdrawal Syndrome (EWS) and Ethanol Consumption During Withdrawal

After 22-d exposure to 6% ethanol in chronic ethanol exposure, ethanol was withdrawn at 9:00. The EWS were then surveyed for 4 min at the 0, 2, 6, 12, 24, 48, and 72 h during ethanol abstinence. Because of short time period, a not full behavioral withdrawal sign was surveyed in this study similar to a previously report (Erden et al., 1999). At each surveying time point, the following behavioral signs in rats were estimated synchronously: body posture, gait, agitation, tail stiffness, tremor, stereotyped behavior, and wet dog shakes. Wet dog shakes and tremors were assessed by incidence. Wet dog shake behavior was considered positive if it occurred at least three times during the observation period. Tremor was determined after lifting rats vertically by the tail: positive was assigned to rats showing clearly distinct forelimb tremor when they were rotated 180°around axis of tail. In the study, grooming, sniffing, head weaving, gnawing, and chewing were observed as major stereotypes behaviors during the ethanol withdrawal. Stereotypic behaviors, abnormal posture and gait, agitation, and tail stiffness were scored using a rating scale (Erden et al., 1999). All ratings of withdrawal signs were scored by an observer who was blind to the treatments on rats.

In IA2BC, after successfully building the model, ethanol was withdrawn at 9:00 h. Then, after 12, 24, 48, or 72 h of a withdrawal period, the amount of alcohol consumed within 6 h after the different alcohol withdrawal time was recorded.

### Stereotaxic Surgery

Animals were anesthetized with ketamine/xylazine (80 mg/5 mg/kg, i.p.), and head was fixed to a stereotaxic frame (RWD Life Science, Shenzhen, China). Ophthalmic ointment was applied to prevent the eyes from drying up and body temperature was maintained using a heated (40°C) pad to prevent hypothermia until awakening. The bregma was exposed, and bilateral guide cannulas (C235G-2.0, 26 gauge; Plastics One) were aimed to the VTA (6.00 mM posterior to the bregma, ± 0.75 mM mediolateral, 7.5 mM ventral to the skull surface), according to Paxinos and Watson (2007). The coordinates for the VTA were identical to those used in a previous study (Guan et al., 2012); hence, it was possible to target the posterior part of this structure, which may be preferentially involved in reward processes and mediation of the reinforcing effects of ethanol (Guan et al., 2012). Immediately after these operations, the rats were placed back to their home cages to recover for at least 1 week.

### Microinjections

After 1 week recovery, subjects were returned to alcohol consumption paradigm; microinjections began when ethanol intakes were stable. BDNF (0.5 μg/μL) (Lu et al., 2004) was dissolved in artificial cerebrospinal fluid (ACSF) that contained the following ingredient (in mM): 250 glycerol, 1.6 KCl, 1.2 NaH_2_PO_4_, 1.2 MgCl_2_, 2.4 CaCl_2_, 25 NaHCO_3_, and 11 glucose (Ye et al., 2006), and then was injected into the bilateral VTA via a 28-gauge internal cannula (RWD Life Science, Shenzhen, China). K252a (1 μg/μL) dissolved in 1% DMSO. The same volume of ACSF was injected into the bilateral VTA in another group of rats as control. Bilateral VTA microinjections (0.5 μL volume per infusion) were performed over 60 s and left in site for post-infusion no <60 s to allow for the drug to spread from the injector tip. Behavioral tests were performed 30 min after the microinjection.

### ELISA Analysis of BDNF

BDNF protein levels were measured by an ELISA test (Vargas-Perez et al., 2009). Briefly, rats were decapitated at different points after withdrawal from ethanol, and their brains were then removed (*n* = 6 per group). A control group of water-treated rats (*n* = 6) was also included. Brains were rapidly extracted, and stored at –80°C. Tissue of VTA was weighed and homogenized in 300 μL of lysis buffer. The homogenates were incubated at 4°C for 30 min and centrifuged at 14,000 g for 30 min. Sandwich-style ELISAs were performed using the Sigma-Aldrich BDNF ELISA kit following the manufacturer’s instructions. BDNF content was interpolated from standard curve runs for each plate. Samples from the treated and control groups were determined in a single run.

### Statistical Analyzes

All data are shown as the mean ± S.E.M. and analyzed using the SPSS 15.0 statistic software (SPSS, Chicago, IL, United States). One-way analysis of variance (ANOVA) was used to make the comparisons between different groups (Li et al., 2019). Significant values obtained using one-way ANOVA were subjected to a Tukey’s multiple comparisons test. A *P*-value of <0.05 was considered statistically significant.

## Results

### Establishment of Rat Models of IA2BC and Chronic Ethanol Exposure

**FIGURE 1:**
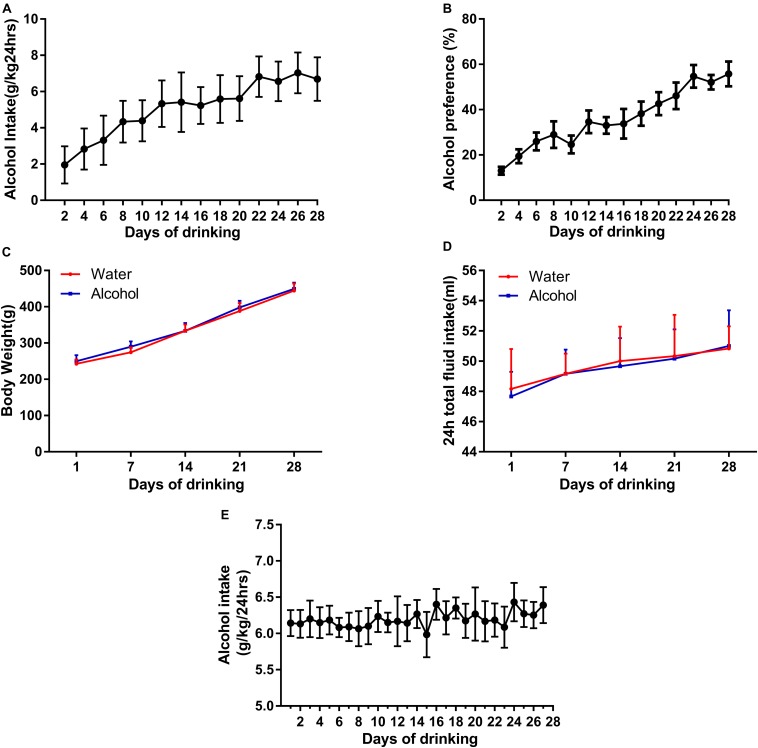
Establishment of two animal models of ethanol-consuming paradigms. We generated a stable intake of alcohol consumption **(A)** and preference **(B)** using IA2BC in Sprague-Dawley rats. There was no significant difference in body weight **(C)** and 24 h total liquid consumption in the ethanol-exposed rats **(D)**. Rats stably consumed an average of 6.39 ± 0.25 g/kg/24 h ethanol in their domestic cages for 28 days in the chronic ethanol exposure paradigm **(E)**. Data are expressed with mean ± standard error of the mean (S.E.M).

First, we used an IA2BC alcohol consumption paradigm to generate individually stable alcohol-consuming rats, which is consistent with report by Carnicella et al. (2014). The rats having access to 20% ethanol showed a robust enhancement in the intake and preference of ethanol across sessions (Figures 1A,B) and reached a stable baseline of ethanol consumption (from 1.95 ± 0.33 to 6.68 ± 0.32 g/kg/24 h) and preference (13.06 ± 1.77 to 55.75 ± 5.5%) after 12 sessions. Rats with ethanol consumption <3.15 g/kg/24 h were excluded in this study. All rats in the IA2BC procedure increased in body weight during establishment of ethanol drinking model, but there was no statistical significance in weight gain between the ethanol and control groups (Figure 1C). Total fluid intake within 24 h did not differ between the alcohol and control groups (Figure 1D).

Because of the fact that ethanol deprivation effects are typically not observed in IA2BC model (Carnicella et al., 2014), in order to observe EWS, we established another model in rats with chronic ethanol exposure. The rats steadily drank an average of 6.39 ± 0.25 g/kg/24 h ethanol in their domestic cages during 28 days in the chronic alcohol exposure paradigm (Figure 1E), similar to description by Turchan et al. (1999).

### Evaluation of Withdrawal Signs and Ethanol Intake

**FIGURE 2:**
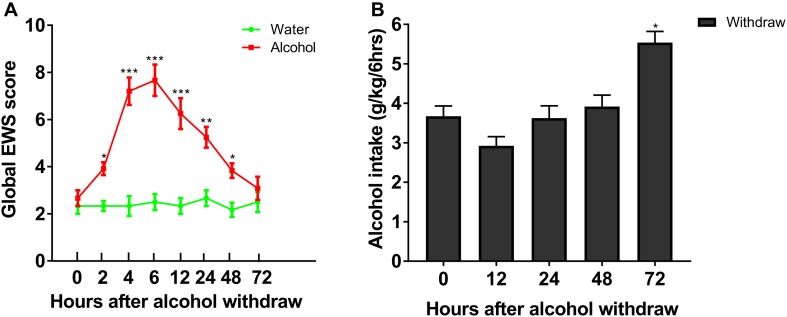
Evaluation of ethanol-related behavior in the two procedures. Global EWS scores and ethanol intake were evaluated after 4 weeks of ethanol consumption in rats with chronic ethanol exposure and IA2BC, respectively. Global EWS scores in rats with chronic ethanol exposure increased at 2, 4, 6, 12, 24, 48, and 72 h after withdrawal from chronic ethanol consumption and peaked at 6 h after withdrawal **(A)**. Firstly, ethanol intake decreased 12 h after withdrawal, and then recovered 24 and 48 h after withdrawal in IA2BC rats. The amount of ethanol intake 72 h after withdrawal exceeded that in the last ethanol consumption session (just before the removal of ethanol) in IA2BC rats **(B)**. Data are expressed with mean ± standard error of the mean (S.E.M). Number of rats per group = 6. *P < 0.05, **P < 0.01, ***P < 0.001 compared to 0 h after withdrawal.

To evaluate withdrawal signs, we assessed scores of stereotypic behaviors, abnormal posture and gait, agitation, and tail stiffness in rats experiencing chronic ethanol exposure. We observed that rats with chronic ethanol exposure showed significant signs at different time point withdrawal (Figure 2A). The global score of EWS escalated from 2.67 ± 0.33 at 0 h to 5.25 ± 0.44 at 24 h (*P* < 0.001), with a peak score around 6 h withdrawal (7.70 ± 0.88, *P* < 0.001), and recovered 72 h withdrawal, indicating an existence of withdrawal severity. Our results implied that the experimental protocol of chronic ethanol exposure can induce physical dependence in rats. In a separate group, rats in IA2BC consumed a significantly higher amount of alcohol 72 h after withdrawal than that at 0 h (in the last ethanol consumption session) (from 3.67 ± 0.26 to 5.54 ± 0.28 g/kg/6 h; *P* < 0.05, Figure 2B). Herein, we found time point diversity in enhanced alcohol intake and evident withdrawal signs after abstinence in these two different alcohol consuming paradigms.

### There Is Negative Correlation Between Expression of BDNF in the VTA and Ethanol-Related Behaviors

**FIGURE 3:**
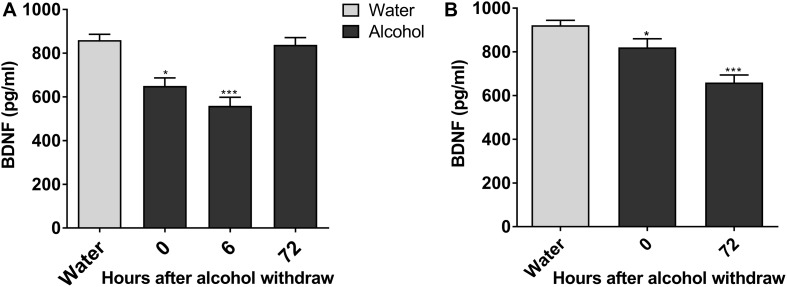
Expression of BDNF in the VTA decreases in alcohol-consuming rats. The expression of BDNF in the VTA in ethanol-consuming rats was analyzed using a BDNF ELISA kit. The level of BDNF expression was lower after abstinence and reached the lowest level 6 h withdrawal in rats with chronic ethanol exposure, and then recovered 72 h after withdrawal to a level similar to the expression in ethanol naive rats **(A)**. BDNF expression levels decreased after withdrawal, and the lowest level of expression was observed 72 h after withdrawal in IA2BC rats **(B)**. Data are expressed with mean ± standard error of the mean (S.E.M). Number of rats per group = 6. *P < 0.05, ***P < 0.001 compared to water group (ethanol naive rats).

Next, we explored molecular mechanisms of enhanced ethanol intake in IA2BC rats and evident withdrawal signs during alcohol withdrawal in rats experiencing chronic ethanol exposure. Considering the key role of BDNF in alcohol addiction and VTA involvement in reward processing (Kauer and Malenka, 2007; Davis, 2008), we studied the expression of BDNF in the VTA when alcohol consumption behavior was evident in these two ethanol consumption patterns. The expression of BDNF in rats 6 h withdrawal from chronic ethanol exposure was lower than that in the control group (559.17 ± 39.38 vs. 860.0 ± 26.52 pg/mL, *P* < 0.001; Figure 3A). Furthermore, BDNF expression declined from 921.83 ± 22.43 to 659.67 ± 34.71 pg/mL in IA2BC rats 72 h after withdrawal (*P* < 0.001; Figure 3B). The time points of low BDNF expression during the withdrawal period paralleled with that of high alcohol intake in IA2BC rats and with that of peak EWS in rats experiencing chronic ethanol exposure respectively.

### 7,8-DHF, a BDNF-Mimicking Small Compound, Reduces Excessive Voluntary Ethanol Intake and EWS

**FIGURE 4:**
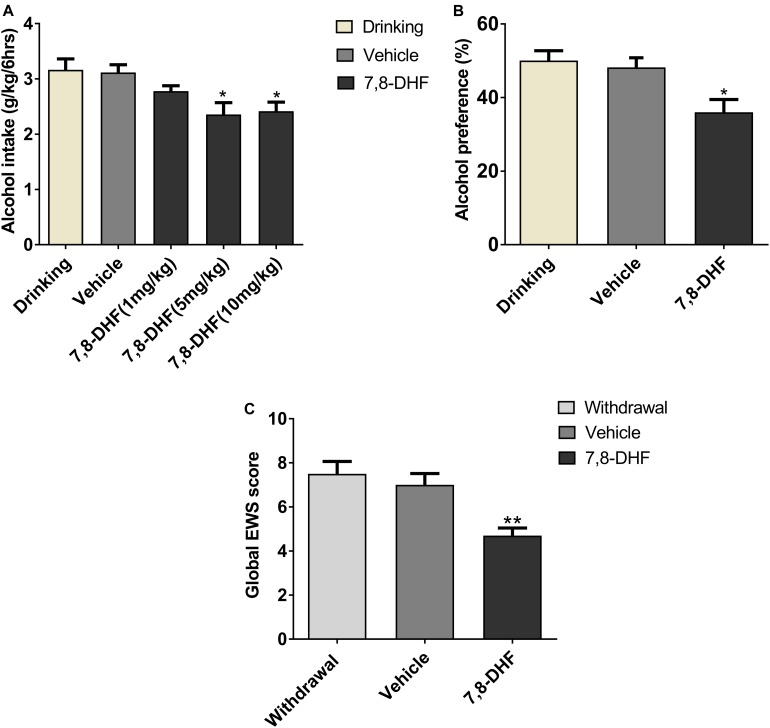
7,8-DHF reduces ethanol-related behaviors. Different doses of 7,8-DHF (1, 5, 10 mg/kg) were intraperitoneally injected in IA2BC rats to identify its effect on ethanol consumption. 7,8-DHF (1 mg/kg) reduced ethanol intake slightly, but 7,8-DHF (5 mg/kg) reduced ethanol intake significantly **(A)**, as observed through comparisons with the vehicle group. 7,8-DHF (5 mg/kg) significantly attenuated ethanol preference in IA2BC rats **(B)**, compared with the vehicle group. 7,8-DHF significantly attenuated global EWS scores 6 h after withdrawal in rats with chronic ethanol exposure **(C)**. Data are expressed with mean ± standard error of the mean (S.E.M). Number of rats per group = 6. *P < 0.05, **P < 0.01, compared to vehicle.

It has been reported that 7,8-DHF (5 mg/kg) can interfere with depression (Zhang et al., 2016) and cognitive function deficits (Tan et al., 2016) effectively in animal models. At first, to test the dose-response effects of 7,8-DHF on alcohol intake, we intraperitoneally injected 7,8-DHF at different doses (1, 5, 10 mg/kg) before examining the amount of intake in IA2BC rats. We showed here that 7,8-DHF 5 mg/kg rather than 1 mg/kg significantly reduced the amount of alcohol intake (from 3.16 ± 0.20 to 2.36 ± 0.21 g/kg; Figure 4A).

To identify if 7,8-DHF could compensate rats for other excessive alcohol-related behaviors, which may be induced by low BDNF expression in VTA, we intraperitoneally injected 7,8-DHF in those group of rats. 7,8-DHF (5 mg/kg) was injected into the abdominal cavity of the subjects before calculating the alcohol preference and EWS. As depicted in Figure 4, 7,8-DHF obviously relieved ethanol preference (36.00 ± 3.47 vs. 50.05 ± 2.67%, *P* < 0.05; Figure 4B), as observed through comparisons with the vehicle-only group of IA2BC rats. In other groups, 7,8-DHF decreased the scores of EWS significantly (4.7 ± 0.34 vs. 7.5 ± 0.56, *P* < 0.01) in rats experiencing chronic ethanol exposure (Figure 4C). These results revealed that injection of exogenous BDNF-mimicking small compound could reduce the ethanol-related behaviors of rats in both ethanol paradigms.

### 7,8-DHF May Affect Alcohol-Related Behavior via TrkB in the VTA

**FIGURE 5:**
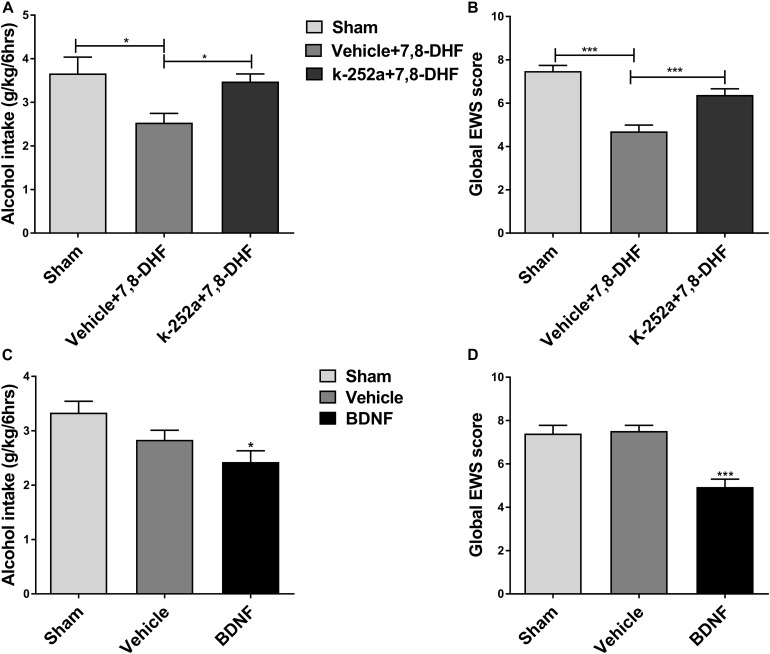
7,8-DHF may reduce ethanol-related behaviors via TrkB in the VTA. K252a (0.5 μg/0.5 μL), a TrkB kinase inhibitor, was microinjected into the VTA before intraperitoneal injection of 7,8-DHF to examine ethanol-related behavior in rats. 7,8-DHF reduced amount of ethanol consumption similar to before in IA2BC rats, which effect was prevented by K252a microinjection 72 h after withdrawal **(A)**. 7,8-DHF significantly attenuated global EWS scores 6 h after withdrawal in rats with chronic ethanol exposure as before, whose effect was antagonized by K252a **(B)**. To further observe if a direct infusion of BDNF into the VTA could mimic 7,8-DHF’s action on ethanol-related behavior, we microinjected BDNF (0.5 μg/μL) into the bilateral VTA in ethanol-consuming rats before detection. We observed a significant reduction in ethanol intake 72 h after withdrawal in IA2BC rats **(C)**, and global EWS scores 6 h after withdrawal in rats experiencing chronic ethanol exposure as well **(D)**, similar to the effects of 7,8-DHF (5 mg/kg) on ethanol behavior. All data are expressed with mean ± standard error of the mean (S.E.M). Number of rats per group = 6. *P < 0.05, ***P < 0.001, compared to vehicle.

Ethanol consumption induces neuroadaptations in the mesolimbic dopaminergic system, which consists of VTA dopamine neurons projecting neural substrates involved in reward processing (Kauer and Malenka, 2007). BDNF and its receptor TrkB are widely distributed in the central nervous system including the VTA and are thought to be involved in neuroplasticity (Chao, 2003). As an important component of the brain’s reward circuitry, the VTA, in general, plays a crucial role in ethanol reinforcement (Gonzales et al., 2004). We hypothesized that if the effects of 7,8-DHF on ethanol-related behavior are involved in TrkB in the VTA, then blocking TrkB in the VTA should antagonize the effects of 7,8-DHF. We therefore microinjected K252a (0.5 μg/0.5μL) (Shirayama et al., 2002), a selective TrkB kinase inhibitor, into the VTA before intraperitoneally administering 7,8-DHF to determine alcohol-related behavior in rats. As showed, microinjection of K252a prevented the reduction in alcohol intake in IA2BC rats and global EWS scores in rats with chronic ethanol exposure. Analysis of these data revealed that treatment with K252a blocked a significant effect of 7,8-DHF by decreasing the amount of alcohol consumption from 2.53 ± 0.21 to 3.48 ± 0.18 g/kg (*P* < 0.05; Figure 5A) and global EWS scores from 4.7 ± 0.29 to 6.38 ± 0.28 (*P* < 0.001; Figure 5B).

If an intraperitoneal injection of 7,8-DHF affects ethanol-related behavior via the activation of TrkB in the VTA indeed, micro-infusion of BDNF into the VTA could mimic the effects of 7,8-DHF. In order to test whether direct injection of BDNF into the VTA could mimic the effects of 7,8-DHF on ethanol-related behaviors, we microinjected BDNF (0.5 μg/μL) (Lu et al., 2004) into the bilateral VTA in ethanol-consuming rats. We observed a significant reduction in alcohol intake from 3.33 ± 0.21 to 2.43 ± 0.21 g/kg (*P* < 0.05; Figure 5C) in IA2BC rats and in withdrawal signs from 7.52 ± 0.26 to 4.93 ± 0.37 (*P* < 0.001; Figure 5D) in rats with chronic ethanol exposure, similar to the effects of 7,8-DHF on alcohol-related behaviors. These results suggest that 7,8-DHF, when administered intraperitoneally, could attenuate alcohol-related behavior via the activation of TrkB in the VTA.

## Discussion

Our study found that 7,8-DHF could attenuate ethanol-related behaviors, including consumption, preference in IA2BC rats, and scores of EWS in rats with chronic ethanol exposure, whose effects were blocked by infusion of K252a, a TrkB antagonist, into the VTA. Directly infusing BDNF into the VTA mimicked the effects of 7,8-DHF on ethanol-related behavior. These results indicated that 7,8-DHF could attenuate ethanol-related behavior via TrkB in the VTA in rats with alcohol consumption.

Continual consumption of alcohol could induce ethanol-dependence and addiction (Majchrowicz, 1975); EWS scores obtained after the withdrawal of chronic alcohol consumption is the most important evidence (Majchrowicz, 1975; Logrip et al., 2015). We found that abstaining from long-term alcohol consumption induces withdrawal signs and increased subsequent alcohol consumption in rats. Consistent with previous report (Li et al., 2019), it was shown in our study that the EWS (including stereotyped behavior, agitation, tail stiffness, abnormal posture, and gait.) score increased at 2 h, and then recovered to a level equivalent to that in the ethanol naïve group at 72 h while it peaked 6 h after alcohol withdrawal in rats with chronic ethanol exposure. In addition, similar to previous reports that abstinence from intermittent alcohol intake induces a higher amount of subsequent ethanol consumption in animals (Miller and Sweatt, 2007; George et al., 2012), here we showed that ethanol intake significantly increased in IA2BC rats 72 h after withdrawal, compared with that in last drinking session.

7,8-DHF, a recently identified selective agonist of TrkB (Jang et al., 2010), can cross the BBB, and studies suggest 7,8-DHF possess potential therapeutic efficacy in various animal models of disease that are relevant to deficient BDNF-TrkB signaling (Jang et al., 2010; Liu et al., 2016). It has been shown that 7,8-DHF demonstrates anti-depressant effects on phenotype and morphological changes in animals induced by lipopolysaccharide administration (Zhang et al., 2014). Additional studies have indicated that a required 5 mg/kg dosage of 7,8-DHF makes it an ideal candidate to study its neuroprotective effects for Alzheimer’s disease (Devi and Ohno, 2012) and age-related cognitive deficits (Zeng et al., 2012). We chose to administer 5 mg/kg 7,8-DHF in the current study, and discovered that 7,8-DHF significantly reduces alcohol intake in IA2BC drinking rats and global EWS scores in rat models of chronic alcohol consumption, respectively. Furthermore, the prior infusion of K252a, a TrkB antagonist, into the VTA antagonized the action of 7,8-DHF on either EWS or ethanol intake, indicating that 7,8-DHF may attenuate alcohol-related behavior via the activation of TrkB in the VTA.

Previous studies have demonstrated that BDNF has an impact on drug abuse, including alcohol (Davis, 2008), which may weaken various phenotypes associated with alcohol consumption through specific brain regions (Pandey et al., 2006). It was reported that the anxiety disorder caused by alcohol withdrawal is related to a decrease in the expression of BDNF (Pandey et al., 2006), and micro-infusion of BDNF in the central nucleus of the amygdala not only reduces ethanol consumption but also reverses the anxiety induced by ethanol withdrawal (Pandey et al., 2006, 2008). Chronic alcohol consumption caused a decrease in BDNF expression in the mPFC of mice (Darcq et al., 2015), and wistar rats sustained alcohol vapor inhalation (3 h/day) for 10 days decreases BDNF level in the hippocampus region (Hauser et al., 2011). Consistent with these findings, in our study, noteworthy lower expression of BDNF was found in the VTA in rats either 6 h withdrawal from chronic alcohol consumption or 72 h withdrawal from IA2BC drinking. However, it should be noted that not all ethanol models produce similar changes in behavior and BDNF expression (Nikulina et al., 2014). Contrary to these reports, others showed that anxiolytic effects of acute ethanol are associated with increased BDNF level in amygdala (Pandey et al., 2008; Moonat et al., 2013), and withdrawal from chronic alcohol consumption increases BDNF expressions in the rat hippocampus and mPFC (Somkuwar et al., 2016). Acute alcohol administration or chronic ethanol drinking increased BDNF expressions in the **dorsal striatum** of mice (McGough et al., 2004). The authors observed that lower expressions of BDNF results in enhanced behavioral responses to alcohol, whereas increases in the levels of BDNF attenuate these behaviors (McGough et al., 2004). Another group also reported that microinjection of BDNF into the dorsolateral striatum attenuates the amount of alcohol intake in Long–Evans rats (Jeanblanc et al., 2009). In this study, we showed that microinjection of BDNF into the bilateral VTA of SD rats attenuates ethanol intake and EWS, similar to the effect of intraperitoneal injection of 7,8-DHF. Taking together, these findings indicate that the BDNF signal might implicate in counteracting neuroadaptations caused by AUD. The diversity of BDNF expression in different neural substrates may differ across brain regions with distinct behavior functions, species, or ethanol delivering methods.

Drugs of abuse can hijack synaptic plasticity mechanisms in the brain circuits of mesolimbic dopamine system (Kauer and Malenka, 2007). Although the circuits in VTA dopamine system implicated in drug abuse have been researched abundantly, it is clear that other brain regions are essential components as well (Kauer and Malenka, 2007). Ventral pallidum projections to hypothalamus and/or subthalamic nucleus are strongly implicated in reinstatement and reacquisition of alcohol seeking (Prasad and McNally, 2016; Prasad et al., 2020). However, detailed mapping of BDNF immunolabeling and mRNA expression have revealed that the VTA contains a medium-to-high density of BDNF expression (Nikulina et al., 2014). It is likely in this study that the mechanisms underlying action of BDNF-TrkB on ethanol-related behaviors may involve in VTA mainly. The expression of BDNF has been associated with increased dopamine metabolism and activity-induced release from midbrain dopaminergic neurons (Nikulina et al., 2014). TrkB receptor dimerization that is activated by BDNF leads to activation of intracellular signaling cascades (Huang and Reichardt, 2001; Nikulina et al., 2014). Chronic alcohol consumption enhances the excitabilities of VTA neurons to acute alcohol treatment, suggesting that neuroadaptation occurs during alcohol drinking (Brodie, 2002). In our study, repeated ethanol consumption induces low expression of BDNF in rat VTA, and microinjection of exogenous BDNF into VTA counteracts EWS or excessive ethanol intake during withdrawal. The mechanisms of BDNF action could be related to synaptic transmission and/or synaptic plasticity in VTA because drugs including ethanol can regulate neuro-circuit function as well as long-term synaptic plasticity in VTA (Kauer and Malenka, 2007; Nugent et al., 2007; Guan and Ye, 2010; Nelson et al., 2018). Importantly, reversing these drug-induced synaptic plasticity may be favorable to the cure for drug addiction including AUD (Kauer and Malenka, 2007). Studies demonstrated that BDNF significantly inhibits the inhibitory post-synaptic currents recorded from rat medial prefrontal cortex as well as supraoptic nucleus (Ohbuchi et al., 2009; Lu et al., 2010). Through TrkB, BDNF treatment results in an increase in the membrane NMDARs and GABA_*A*_Rs in rat hippocampal neurons (Elmariah et al., 2004). On the other hand, BDNF acts at presynaptic terminals to increase glutamate release in hippocampal slice cultures possibly by facilitating vesicle mobilization and/or fusion at presynaptic active zones (Tyler et al., 2002). Not only synaptic transmission, but also synaptic plasticity may be involved in the mechanism of BDNF actions on ethanol-related behaviors in the current study, which is supported by following discoveries. Excitatory synapses onto dopamine neurons in the VTA of the rat midbrain become highly susceptible to potentiation by weak presynaptic stimuli after withdrawal from repeated cocaine exposure requiring endogenous BDNF-TrkB signaling (Pu et al., 2006). We previously reported that LTP_*GABA*_ in VTA was blocked 24 h after intraperitoneal injection of ethanol (Guan and Ye, 2010), but the detailed mechanism remains unknown. One report indicates that ethanol exposure potently inhibits BDNF-dependent LTP_*GABA*_ in rat CA3 hippocampus (Zucca and Valenzuela, 2010). Another report suggests that BDNF could contribute the induction of long-term potentiation of inhibitory synapses in visual cortical pyramidal neurons through TrkB (Inagaki et al., 2008). Therefore, BDNF may contribute to the development of LTP_*GABA*_ in VTA, which is inhibited by ethanol. Suppose that BDNF contributed induction of LTP_*GABA*_ in VTA, and then the low expression of BDNF induced by repeated ethanol exposure may mediate the impairment of LTP_*GABA*_. If so, it is possible that infusion of exogenous BDNF into VTA rescued the impaired LTP_*GABA*_ by ethanol so that BDNF/7,8-DHF might attenuate rewarding effects of ethanol through indirect inhibition on dopaminergic neurons in VTA. The exact role of BDNF in development of LTP_*GABA*_ in VTA in rats still requires future investigation.

## Conclusion

In conclusion, these results indicate that BDNF-TrkB signals in the VTA are implicated in expression of ethanol-related behaviors. Administration of exogenous 7,8-DHF attenuated alcohol-related behavior in rats via TrkB in the VTA. Our findings suggest BDNF-TrkB in VTA is a part of regulating signals for opposing neural adaptations in AUD, and 7,8-DHF may serve as a potential candidate for treating alcoholism.

## Data Availability Statement

All datasets generated for this study are included in the article/supplementary material.

## Ethics Statement

The animal study was reviewed and approved by Institutional Animal Care and Use Committee of the Mudanjiang Medical University.

## Author Contributions

Y-ZG designed the research. X-XL, L-LZ, TY, NW, QG, and Y-MX conducted the experiments. X-XL, TY, and XL performed the data analysis. TY, X-XL, and Y-ZG contributed to the writing of the manuscript. X-FZ and Y-ZG supervised the project.

## Conflict of Interest

The authors declare that the research was conducted in the absence of any commercial or financial relationships that could be construed as a potential conflict of interest.
